# Fungi under fire: diagnostic capacities and antifungal availability in Peruvian healthcare facilities

**DOI:** 10.1128/spectrum.02020-24

**Published:** 2025-06-20

**Authors:** Julio Maquera-Afaray, Luis E. Cuéllar, Oliver A. Cornely, Jon Salmanton-García, IFI Diagnostic and Treatment Capacity Collaborator Team Peru

**Affiliations:** 1 Instituto de Investigación en Ciencias Biomédicas, Universidad Ricardo Palma, Lima, Peru; 2 Instituto Nacional de Enfermedades Neoplásicas 74483, Lima, Peru; 3 Universidad Nacional Federico Villarreal 125819, Lima, Peru; 4 Institute of Translational Research, Cologne Excellence Cluster On Cellular Stress Responses in Aging-Associated Diseases (CECAD), Faculty of Medicine and University Hospital Cologne, University of Cologne 14309, Cologne, Germany; 5 Department I of Internal Medicine, Center for Integrated Oncology Aachen Bonn Cologne Duesseldorf (CIO ABCD) and Excellence Center for Medical Mycology (ECMM), Faculty of Medicine and University Hospital Cologne, University of Cologne 14309, Cologne, Germany; 6 German Centre for Infection Research (DZIF), Partner Site Bonn-Cologne, Cologne, Germany; 7 Clinical Trials Centre Cologne (ZKS Köln), Faculty of Medicine and University Hospital Cologne, University of Cologne 14309, Cologne, Germany; University of Lagos, Lagos, Nigeria

**Keywords:** invasive fungal infections, Peru, diagnostic capabilities, mycology laboratories, antifungal treatments, microscopy, species identification, antibody detection, antigen detection, therapeutic drug monitoring

## Abstract

Invasive fungal infections (IFI) are a significant global health issue, affecting an estimated 7 million people annually, with around 3 million deaths. In Peru, IFIs are estimated to affect about 2% of the population. Underdiagnosis due to the limited sensitivity of diagnostic tests underestimates the true burden. This study evaluates the diagnostic capabilities of mycology laboratories and the availability of antifungal treatments in Peruvian healthcare facilities to identify gaps and improve IFI management. An observational, cross-sectional study was conducted online targeting physicians involved in IFI management across multiple centers in Peru, from April 2023 to April 2024. The survey covered institutional profiles, incidence and perceived relevance of IFI, diagnostic tools, and access to antifungal drugs. Fifty-four centers from 21/24 departments (Peruvian term for regions) in Peru participated. All centers reported a low to moderate IFI incidence. *Candida* spp. was the most concerning pathogen (93%), followed by *Aspergillus* spp. and *Cryptococcus* spp. (57% each). Diagnostic methods like microscopy were universally used (100%), while culture-based diagnosis was available in 90% of centers. Access to advanced diagnostics for species identification varied, with better availability in the capital (91%) compared to regions (64%). Antibody detection tests were available in 30% of centers, mostly in the capital area. Antigen detection tests were available in 46% of institutions, with significant regional disparities. Imaging techniques were widely used, but surgical access varied. Triazoles were the most accessible antifungals (96%), while echinocandins and therapeutic drug monitoring (TDM) were significantly limited (37% and 2%, respectively). The study highlights disparities in the availability of advanced diagnostics and antifungals in Peru. Despite universal use of microscopy, access to species identification, antibody, and antigen detection tests is limited outside the capital. Ensuring equitable access to these resources and implementing therapeutic drug monitoring are crucial for improving IFI management in Peru.

**IMPORTANCE:**

Invasive fungal infections are a critical yet often underrecognized public health issue, particularly in countries with diverse climates like Peru. Limited access to advanced diagnostic tools and antifungal treatments creates significant barriers to effective management, contributing to underdiagnosis and delayed care. Our study provides an in-depth evaluation of current diagnostic capabilities and drug availability for IFI across Peru, uncovering geographic disparities and resource gaps that affect patient outcomes. This research highlights the urgent need for policy reforms aimed at enhancing laboratory infrastructure and access to antifungal therapies, ultimately improving IFI management and reducing mortality in Peru and similar regions globally.

## INTRODUCTION

Currently, invasive fungal infections (IFI) constitute a growing public health concern globally, with an estimated 6.55 million people affected each year worldwide, and around 2.55 million deaths directly attributable (1, 2). In Peru, IFIs were estimated to represent approximately 1.9% of the total Peruvian population. However, this result may underestimate the actual IFI burden in Peru, due to underdiagnosis from the use of diagnostic tests with limited sensitivity (3). In addition, certain conditions favor the presence of IFI in Peru, such as endemic mycoses (4–11) due to the country’s geographical climate heterogeneity (Fig. S1), such as histoplasmosis (4, 5, 8, 9, 11, 12), paracoccidioidomycosis (4, 5, 9, 11), and sporotrichosis (4, 7, 9, 11, 13). These endemic IFIs are opportunistic mycoses related to human immunodeficiency virus (HIV), primarily due to late diagnosis and advanced disease (such as cryptococcal meningitis) (1, 3, 8, 14, 15); and the emergence of rare fungal infections related to non-HIV immunocompromised hosts, such as onco-hematological patients and organ transplant recipients (11, 16–27).

Recently, the World Health Organization (WHO) selected and prioritized fungal pathogens that impact human health globally, with one of its purposes being to promote health policy interventions that improve access to quality mycological diagnostics, consequently promoting rational use of antifungals (28). Studies have been conducted in different regions of the world on the diagnostic capabilities of mycology laboratories and access to antifungal medications, with variable results differing between regions (6, 29–38). However, existing information in Latin American countries is scarce (6, 31). In 2019, a study included different Latin American countries; however, most participating sites were from Brazil, with only one center from Peru (6).

The objective of this study was to evaluate the present state of diagnostic capabilities of mycology laboratories and the availability of antifungal treatments in healthcare facilities in Peru, with the purpose of identifying gaps to inform improvement strategies in the management of IFI.

## MATERIALS AND METHODS

**Fig 1:**
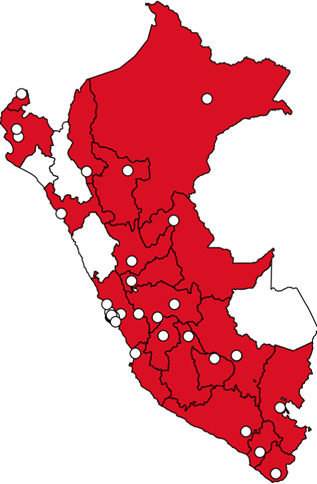
Map of participating institutions per department (Peruvian term for regions). Departments (Peruvian term for regions) with participating institutions are colored in red. Departments (Peruvian term for regions) whose participating centers have not been included are colored in white. If more than one participating center is from the same location, a single circle is pictured. Participating institutions per department (Peruvian term for regions): Lima (*n* = 20/54, 37.0%); Callao and Junín (*n* = 4/54, 7.4% each); La Libertad and Piura (*n* = 3/54, 5.6%, each); Cusco, Huánuco, Loreto, and Pasco (*n* = 2/54, 3.7%, each); and Amazonas, Apurímac, Arequipa, Ayacucho, Huancavelica, Ica, Moquegua, Puno, San Martín, Tacna, Tumbes, and Ucayali (*n* = 1/54, 1.9%, each).

An observational, cross-sectional study was conducted across multiple Peruvian centers via an online survey (Fig. 1). This study targeted physicians who are actively involved in the management of IFI. The participating centers in this survey were selected using a combination of convenience sampling and specific inclusion criteria aimed at ensuring a diverse representation of institutions. Specifically, we aimed to include centers that varied in terms of geographical location, size, and the type of patient populations served. This approach was intended to capture a comprehensive overview of the current practices and challenges in diagnosing IFI across different healthcare settings. The data collection period spanned from April 2023 to April 2024.

The survey was disseminated, and participants were invited through various channels, including social networks, the internal group networks of the Peruvian Society of Infectious and Tropical Diseases (SPEIT, https://speit.pe/), email, and telephone. The questionnaire used in this study, which can be found in Table S1, has been utilized in previous research (6, 29, 31–34, 36–38). It covers a range of topics, including (i) the profile of the institution, (ii) perceptions about the incidence and perceived relevance and concern of IFI in the institution, (iii) microscopy, (iv) culture and identification of fungi, (v) serology, (vi) antigen detection, (vii) molecular assays, (viii) therapeutic drug monitoring (TDM), and (ix) access to antifungal drugs. Efforts were made to limit responses to a single participant per center whenever possible to ensure data consistency. However, in cases where multiple respondents from the same center contributed, responses were harmonized by consolidating them into a single data set per center. This process involved cross-referencing answers to avoid duplication and ensure that each center’s data was accurately represented.

The collected data were presented as frequencies and percentages. Denominators varied according to the number of responses received for each specific variable, reflecting the number of participants who responded for each test. Proportions were displayed in contingency tables and compared using Fisher’s exact test for variables with at least one cell with an expected value of 5, and the X² test for variables with all cells with an expected value of >5, as appropriate. Comparisons were made based on several factors: (i) geographical distribution of the centers (Lima and Callao [capital area, urban] and the rest of the country [rural]), (ii) climatic distribution (coast line, highlands, and rainforest, Fig. S1), (iii) healthcare provider (ESSALUD [Seguro Social de Salud—Social Health Insurance, aimed at formal workers, generally with a better economic status and budget], MINSA [Ministerio de Salud—Ministry of Health, serving people with informal or independent jobs, with resources according to their level of wealth], and the Peruvian Army associated institutions), and (iv) admission of high-risk immunocompromised patients (institutions managing hematopoietic stem-cell transplantation or solid transplantations versus those not). Statistical significance was considered for *P* values < 0.05. The statistical analysis was performed using SPSS v27.0 (SPSS, IBM Corp., Chicago, IL, United States).

## RESULTS

A total of 54 centers from 21 out of 24 departments (Peruvian term for regions) in Peru participated in this study. We observed that all centers (*n* = 54/54, 100%) determined their incidence of IFI to be low to moderate. Nevertheless, statistically significant differences were observed between those centers with high-risk patients and those without (*P* = 0.001) (Table 1; Fig. 1; Table S2).

**TABLE 1:** Access to diagnostic tools of participating institutions in Peru^*a*^

Parameter	Value	
	*N*	%
Estimated IFI incidence		
Very low	26/54	48.1
Low	17/54	31.5
Moderate	11/54	20.4
High	0/54	0.0
Very high	0/54	0.0
Microscopy	54/54	100.0
Microscopy is used if IFI is suspected		
Never	14/54	25.9
Almost never	17/54	31.5
Sometimes	11/54	20.4
Almost always	6/54	11.1
Always	6/54	11.1
Calcofluor white	1/22	4.5
Giemsa stain	39/49	79.6
China/India ink	47/52	90.4
KOH	44/50	88.0
Silver stain	11/36	30.6
Fluorescence dyes	3/54	5.6
Culture	44/49	89.8
Blood culture if fungemia is suspected	29/49	59.2
Available media for fungal culture		
Agar Niger	4/26	15.4
Chromogen agar	8/35	22.9
Lactrimel agar	1/27	3.7
Potato agar	5/30	16.7
Sabouraud	38/44	86.4
Sabouraud + chloramphenicol	12/29	41.4
Sabouraud + gentamicin	8/26	30.8
Selective agar (chloramphenicol + gentamicin)	4/24	16.7
Available tests for species identification	37/49	75.5
Semi-automated identification kit	26/45	57.8
Automated identification system	28/40	70.0
DNA sequencing	2/47	4.3
MALDI-TOF-MS	1/46	2.2
Antifungal susceptibility	27/49	55.1
CLSI	11/40	27.5
EUCAST	1/38	2.6
Gradient strip test	5/36	13.9
Semi-automated antifungal susceptibility testing system	13/39	33.3
Antibody detection	14/47	29.8
*Aspergillus* spp.	9/46	19.6
Onsite	1/9	11.1
Outsourced	8/9	88.9
*Candida* spp.	9/46	19.6
Onsite	3/9	33.3
Outsourced	6/9	66.7
*Histoplasma* spp.	13/46	28.3
Onsite	1/13	7.7
Outsourced	12/13	92.3
*Paracoccidioides* spp.	10/46	21.7
Outsourced	10/10	100.0
Antigen detection	21/46	45.7
*Aspergillus* GM, any	11/46	23.9
*Aspergillus* GM (ELISA)	10/44	22.7
Onsite	3/10	30.0
Outsourced	7/10	70.0
*Aspergillus* GM (LFA)	8/44	18.2
Onsite	1/8	12.5
Outsourced	7/8	87.5
*Aspergillus* GM (LFD)	6/45	13.3
Outsourced	6/6	100.0
*Candida* antimannan	7/44	15.9
Outsourced	7/7	100.0
*Cryptococcus* mannan, any	19/45	42.2
*Cryptococcus* (LAT)	19/45	42.2
Onsite	8/19	42.1
Outsourced	11/19	57.9
*Cryptococcus* (LFA)	11/44	25.0
Onsite	4/11	36.4
Outsourced	7/11	63.6
*Histoplasma* antigen	13/44	29.5
Outsourced	13/13	100.0
Beta-d-glucan	8/45	17.8
Outsourced	8/8	100.0
Molecular tests	10/44	22.7
*Aspergillus* PCR	7/44	15.9
Onsite	2/7	28.6
Outsourced	5/7	71.4
*Candida* PCR	7/44	15.9
Onsite	1/7	14.3
Outsourced	6/7	85.7
*Pneumocystis* PCR	5/44	11.4
Outsourced	5/5	100.0
Mucorales PCR	5/44	11.4
Outsourced	5/5	100.0
Therapeutic drug monitoring	1/46	2.2
Posaconazole	1/46	2.2
Onsite	1/1	100.0
Voriconazole	1/46	2.2
Onsite	1/1	100.0
Imaging procedures		
CT	39/46	84.8
PET CT	2/46	4.3
MRI	14/46	30.4
PET MRI	2/46	4.3
Ultrasound	43/46	93.5
X ray	37/46	80.4
Surgery	18/31	58.1

^*a*^ CLSI, Clinical and Laboratory Standards Institute; CT, computed tomography; DNA, deoxyribonucleic acid; ELISA, enzyme-linked immunosorbent assay; EUCAST, European Committee on Antimicrobial Susceptibility Testing; GM, galactomannan; IFI, invasive fungal infection; KOH, potassium hydroxide; LAT, latex agglutination test; LFA, lateral flow assay; LFD, lateral flow device; MALDI-TOF-MS, matrix-assisted laser desorption/ionization-time of flight mass spectrometry; MRI, magnetic resonance imaging; PCR, polymerase chain reaction; PET, positron emission tomography; spp., species.

**Fig 2:**
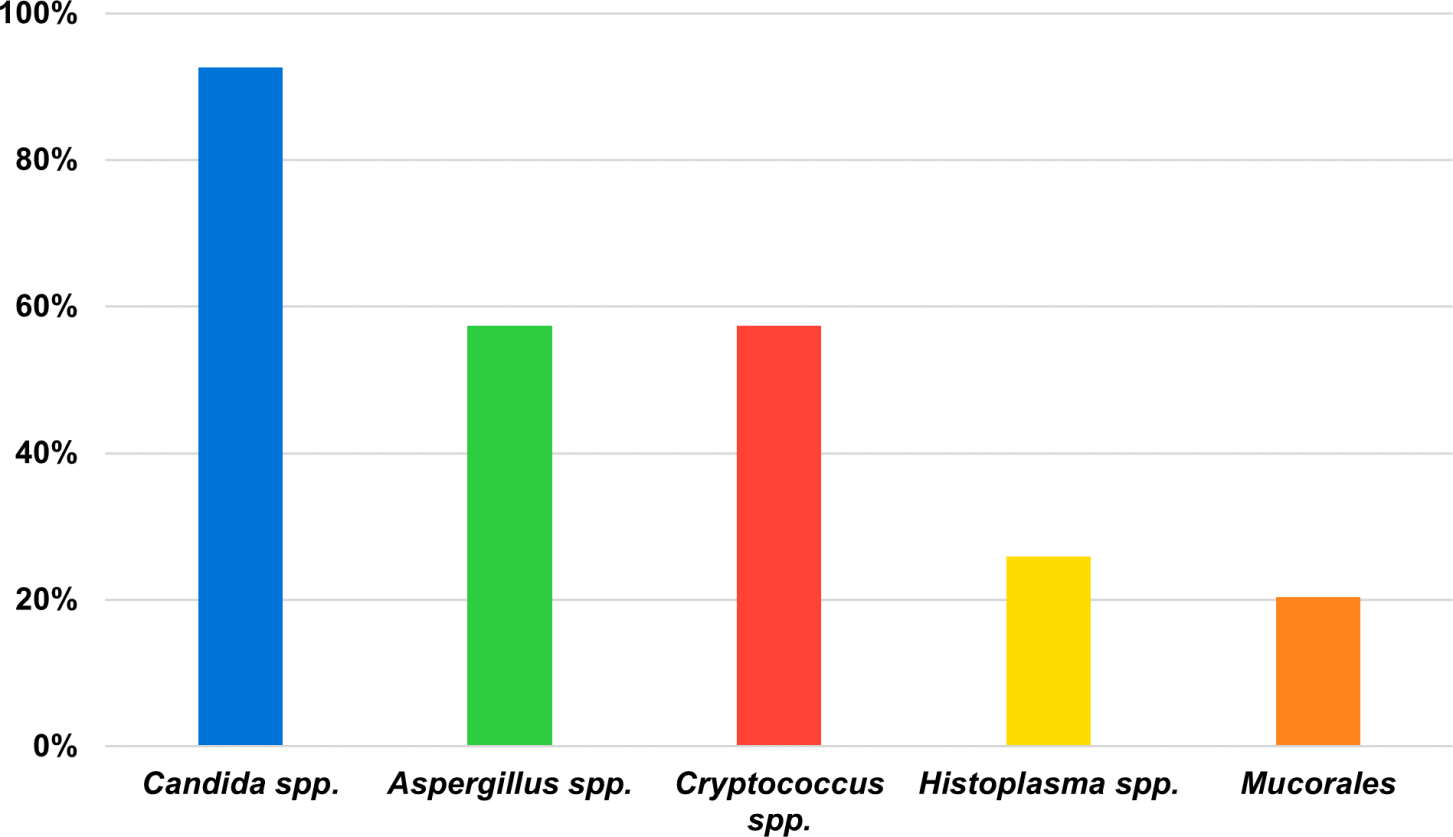
Fungal pathogens described as of most relevance in Peru.

*Candida* spp. was considered the most concerning fungus, reported from 92.6% of the centers (*n* = 50/54). Following this, *Aspergillus* spp. and *Cryptococcus* spp., each was reported from 57.4% of the centers (*n* = 31/54). Statistically significant differences were observed in the classification of fungi by their relevance for *Cryptococcus* spp. (considered of particular concern in MINSA centers [*n* = 23/33, 69.7%] than in ESSALUD centers [*n* = 6/19, 31.6%], *P* = 0.008) and in Mucorales, more frequently reported in the capital area (*n* = 8/24, 33.3%) than in provinces (*n* = 3/30, 10.0%, *P* = 0.046). Of note, Mucorales were reported as a pathogen to consider only on the coast (11/36, *P* = 0.024) (Table 1; Fig. 2; Table S2).

Microscopy was shown as a universally used diagnostic tool when IFI is suspected, with 100% use in all registered centers. The most frequent microscopic methodologies were China/India ink (*n* = 47/52, 90.4%), potassium hydroxide (KOH, *n* = 44/50, 88.0%), and Giemsa stain (*n* = 39/49, 79.6%) (Table 1; Table S2).

Culture-based diagnosis was available in 89.8% of the centers (*n* = 44/49), observing a variable availability of fungal species identification tests, with 75.5% of the centers (*n* = 37/49) with access to some form of identification test, mostly with automated (*n* = 28/40, 70.0%) and semi-automated tests (*n* = 26/45, 57.8%). Access to species identification tests was significantly higher in the capital area (*n* = 19/21, 90.5%) compared to the rest of the country (*n* = 18/28, 64.3%, *P* = 0.047). Although susceptibility tests were available in a little more than half of the evaluated laboratories (*n* = 27/49, 55.1%), there was variability in the reference standards used, with most of these centers using a semi-automated antifungal susceptibility testing system (*n* = 13/39, 33.3%) (Table 1; Table S2).

Antibody detection tests were available in 14/47 (29.8%) centers, mostly in the capital area (capital area *n* = 11/20, 55.0%; rest of the country *n* = 3/27, 11.1%, *P* = 0.003). Accessibility to tests for the detection of *Histoplasma* spp. was the highest (*n* = 13/46, 28.3%). A total of 21/46 (45.7%) institutions reported access to antigen detection tests, also with significant differences between the capital area (*n* = 14/21, 66.7%) and provinces (*n* = 7/25, 28.0%), *P* = 0.017. The most frequent were those for *Cryptococcus* spp. (*n* = 19/45, 42.2%) and *Aspergillus* spp. (*n* = 11/46, 23.9%). Almost one-fifth of the centers (*n* = 8/45, 17.8%) reported access to beta-d-glucan. PCR diagnosis was reported as accessible in 10/44 (22.7%) centers (Table 1; Table S2).

Imaging procedures, such as ultrasound (*n* = 43/46, 93.5%), computerized tomography (CT, *n* = 39/46, 84.8%), or X-ray (*n* = 37/46, 80.4%), were widely reported in the diagnosis and follow-up of IFI. In total, surgery is performed in 58.1% (*n* = 18/31) of the centers, with statistically significant differences between capital and provinces (*P* = 0.010), depending on the climatic region (*P* = 0.024) or the healthcare provider (*P* = 0.014) (Table 1; Table S2).

**Fig 3:**
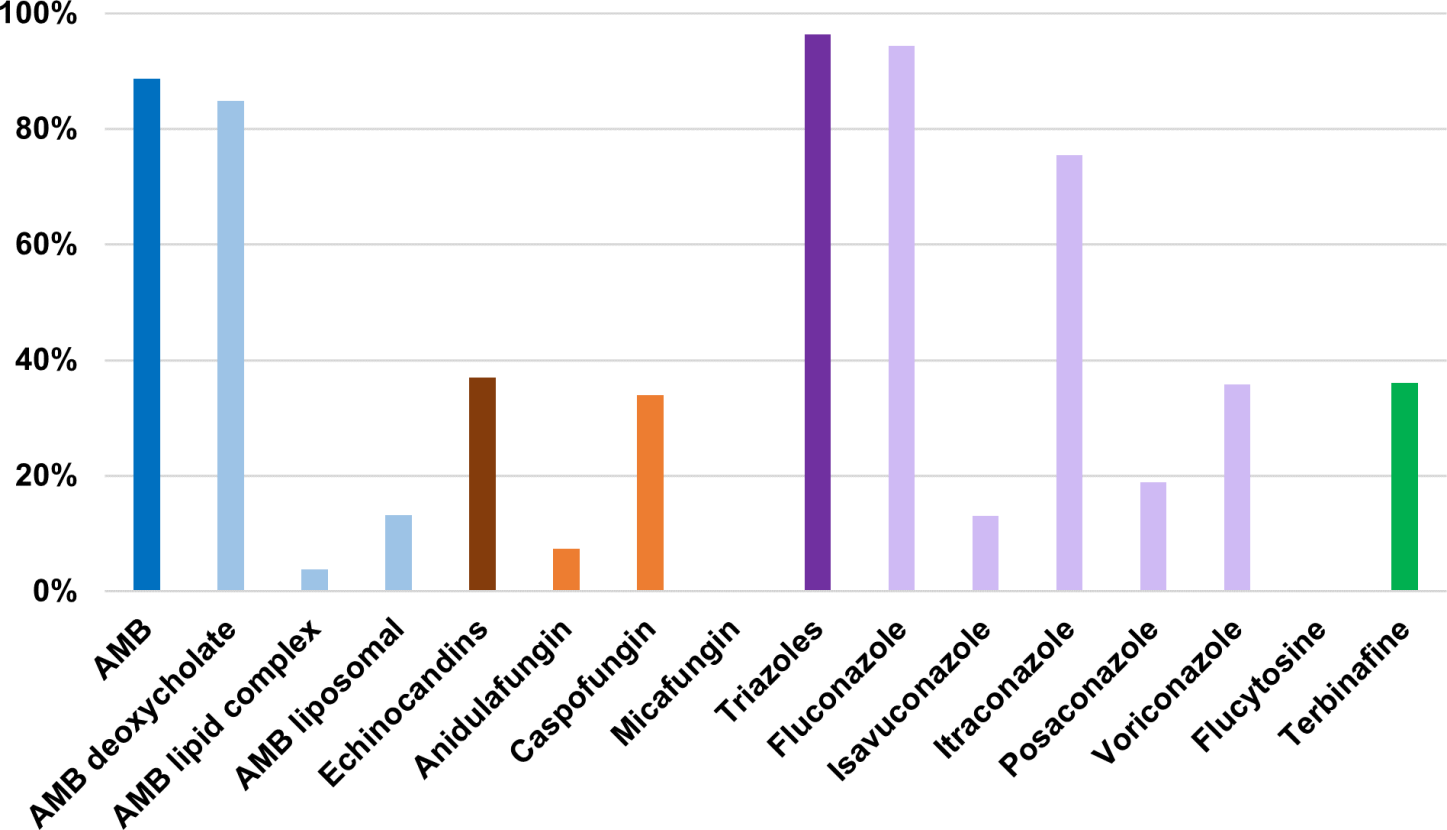
Described access to antifungals in Peru.

In addition, the data revealed a wide availability of different antifungals. Triazoles were the most accessible, available in 96.3% (*n* = 52/54) of the centers, especially fluconazole, present in 94.4% (*n* = 51/54) of the centers. Among the mold-active antifungals, the most frequent was itraconazole (*n* = 40/53, 75.5%). Furthermore, 88.7% (*n* = 47/53) of the centers reported access to at least one formulation of amphotericin B, mainly deoxycholate (*n* = 45/53, 84.9%). Access to any echinocandin was limited to 37.0% (*n* = 20/54), while it was 36.0% (*n* = 18/50) for terbinafine. Statistically significant differences were observed in access to certain specific antifungals in all comparisons performed (capital versus rest of the country, climatic region, health provider, admission of high-risk patients for IFI). Only 1/46 center (2.2%) had access to TDM (Table 1; Fig. 3; Table S2).

## DISCUSSION

The study provides a thorough analysis across 54 centers from 21 out of 24 departments (Peruvian term for regions) in Peru, offering a robust sample for evaluating the incidence and management of IFI. While traditional diagnostic methods are widely used, access to advanced diagnostics and antifungal medications varies, indicating resource disparities. Therapeutic drug monitoring is notably lacking.

All centers report a low to moderate incidence of IFI, although significant differences emerge between centers treating high-risk patients and those that do not. In fact, centers with high-risk patients tend to be more vigilant about the potential appearance of IFI. However, despite previous efforts, there is still a need for enhanced surveillance systems to accurately determine which is the true incidence of IFI in Peru and its associated burden (3).

*Candida* spp. are identified as the most concerning pathogens by the majority of the centers, aligning with global trends (6, 28, 32, 33, 37). Nevertheless, our study reveals notable differences in the perceived relevance of *Cryptococcus* spp. and Mucorales based on the healthcare provider and the geographic location of the analyzed institutions. For instance, *Cryptococcus* spp. is seen as more concerning in MINSA centers (69.7%) compared to ESSALUD centers (31.6%), reflecting differences in patient demographics and conditions treated: ESSALUD primarily caters to formal workers, typically those with stable economic status and financial resources. By contrast, MINSA focuses on serving individuals with informal or independent employment, adjusting its resources based on their wealth level. In parallel, Mucorales is more frequently reported in the capital area (33.3%) versus the other parts of the country (10.0%), likely due to the higher prevalence of mucormycosis among highly immunocompromised patients who are more often treated in urban areas like Lima, the capital of Peru.

Microscopy is the cornerstone of IFI diagnosis in the depicted institutions, as it is universally employed across all centers. Traditional methods such as China/India ink, potassium hydroxide, and Giemsa stain are widely used, underscoring their cost-effectiveness despite limitations in sensitivity and specificity. China/India ink staining, crucial in Peru for diagnosing meningeal cryptococcosis in HIV patients (39, 40), is accessible in most centers, despite its lower sensitivity compared to *Cryptococcus* spp. antigen detection tests (41). While microscopy remains essential and widely available, as it is in other global regions and countries (6, 29, 31–34, 36, 37), additional diagnostic methods are needed to enhance accuracy.

Culture-based diagnosis, which remains the gold standard for identifying specific pathogens and performing antifungal susceptibility tests (42), is available in most laboratories. However, there is a disparity in access to species identification tests, with the capital area better equipped (90.5%) compared to other regions (64.3%). This highlights the need to standardize homogeneous access to culture and species identification tests across the whole country. Advanced methods like matrix-assisted laser desorption/ionization time-of-flight mass spectrometry (MALDI-TOF-MS) are rare, with only one center (1.9%) having this capability. This is in stark contrast to higher usage in countries such as Austria (100.0%) (34), Germany (90.7%) (43), Italy (81.6%) (36), Portugal (62.5%) (38), Argentina (53.0%) (31), or Hungary (47.1%) (29).

Antibody detection tests are available in one-third of the centers, primarily in the capital area (55.0%) compared to the rest of the country (11.1%). Antigen detection tests are more common, available in half of the surveyed institutions, again with a significant disparity between the capital and other regions. The most frequently available tests target *Cryptococcus* spp. and *Aspergillus* spp. No center in Peru reports having the *Histoplasma* spp. antigen test onsite (one-third outsourced), and only one center has the serological test for histoplasmosis onsite (one-third outsourced), despite its importance in diagnosing this locally endemic fungal infection (5, 8). Thus, enhancing the availability of antibody and antigen detection tests is crucial for early and accurate IFI detection (25, 44–47). In parallel, outsourcing diagnostic tests for IFI affects cost, accessibility, and timeliness, all of which are crucial for patient outcomes. While it can reduce initial expenses by eliminating the need for in-house equipment and staff, outsourced tests often incur higher fees and lead to cumulative costs that may surpass local diagnostics (48). In rural areas, outsourcing frequently delays access to critical results—often taking days or weeks—when rapid diagnosis is essential for effective management. In addition, these tests can complicate result interpretation for local clinicians unfamiliar with external methodologies, hindering timely treatment decisions for IFI.

Of note, Peru has a National Reference Laboratory, part of the National Institute of Health (INS), which supports diagnosis with mycological tests such as gel immunodiffusion for *Histoplasma* spp., *Paracoccidioides* spp., and *Aspergillus* spp., latex agglutination for *Cryptococcus* spp., as well as culture, typing, and fungal susceptibility testing (49).

Imaging techniques, including ultrasound, CT scans, and X-rays, are widely used for diagnosing and monitoring IFI. However, accessibility varies significantly by location and healthcare provider. Ensuring equitable and uniform access to imaging facilities is essential for comprehensive IFI management, along with training and resources to improve imaging capabilities, especially in non-capital regions.

Regarding access to antifungal medications, the study reveals significant differences between centers, with cost being considered a major limiting factor in some areas (50). Access to lipid formulations of amphotericin B (13.2%), preferred for their efficacy and safety over amphotericin B deoxycholate (84.9%), is limited despite being first-line treatments for IFI like meningeal cryptococcosis (16) and mucormycosis (18). This contrasts with neighboring Argentina and Brazil (6, 31), where liposomal amphotericin B is available in 73% and 43% of centers, respectively. In Europe (33), lipid formulations of amphotericin B (range 78%–88%) are more common than amphotericin B deoxycholate (range 18%–41%).

According to our study, fluconazole, which is recommended for treating invasive candidiasis caused by *Candida albicans* and other susceptible yeast species (17, 19, 22, 23, 26), is available in nearly all centers (94.4%), followed by itraconazole (75.5%), widely used in the treatment of endemic mycoses (11). However, other triazoles active against filamentous fungi like voriconazole (35.8%), posaconazole (18.9%), or isavuconazole (13.0%) are significantly less accessible. This limited access is similar to what is described in African countries (37), where access to isavuconazole, for instance, was less than 5%. Closer to Peru, in Argentina, isavuconazole availability reached 33% (31), while in European countries (33), access to isavuconazole ranged from 61% to 80%, posaconazole from 77% to 84%, and voriconazole from 89% to 90%.

With the emergence of non-*albicans Candida* species resistant to triazoles (51), echinocandins (37.0%) have become essential for treating invasive candidiasis, with evidence supporting their use to improve survival and therapeutic success. About one-third of the participating centers report access to at least one echinocandin, primarily caspofungin (34.0%) and anidulafungin (7.4%), with none having access to micafungin. In Brazilian centers (6), echinocandin access was micafungin 48%, anidulafungin 32%, and caspofungin 16%. Similarly, in Argentine centers (31), 53% had access to at least one echinocandin. In Europe (33), access was 89%, in Asia-Pacific 72% (32), and in Africa^37^ between 5% and 23%, depending on the specific echinocandin.

The situation with flucytosine, despite its classification as an essential drug by the World Health Organization (WHO) (52), is particularly concerning. This antifungal is especially useful in treating meningeal cryptococcosis alongside liposomal amphotericin B (16). According to our results, it is unavailable in Peru and not widely available in Latin America either (6). In Brazil (6), only 18% of centers have access to this medication. This contrasts with other regions, such as Europe, where access reaches 50% (33), the Asia/Pacific region with 43% (32), and Africa with 28% (37). Yet, even in regions with broader availability, access limitations have been reported (53, 54).

TDM is essential for optimizing antifungal therapies like voriconazole and posaconazole, yet only one center in Peru performs this critical test. TDM ensures drug concentrations are maintained within therapeutic ranges, allowing for individualized dosing based on factors such as patient weight and renal function. Without TDM, reliance on standard dosing can lead to treatment failures or increased adverse events, negatively impacting patient outcomes, particularly in those with azole-resistant strains. Limited access to TDM can worsen infections and heighten mortality risks; thus, discussing TDM access in IFI management is vital, emphasizing its importance in clinical guidelines and showcasing outcomes with versus without TDM to highlight its benefits.

One limitation of the study is the potential bias introduced by the non-inclusion of several centers, particularly those from cities in the interior of Peru, despite the participation of a considerable number of centers from different regions. In addition, many participants were from centers with specialists in infectious and tropical diseases, which could skew the findings. Another limitation is the variety of denominators reported for different variables, resulting from non-responses from participants. This variation may impact the interpretation of the findings, as not all participants answered every question, leading to differing sample sizes for each reported frequency. We also acknowledge that the study relies on self-reported data from healthcare institutions, which may introduce bias due to differences in respondents’ knowledge, willingness to participate, and institutional capacity. This limitation should be taken into account when interpreting the results.

To address the needs identified in the current status highlighted by the study, potential policy changes could focus on increasing funding and resources for diagnostic infrastructure in underserved areas. This might involve creating partnerships between public health agencies and local healthcare providers to improve access to advanced diagnostic tools and training. Furthermore, launching targeted outreach initiatives to raise awareness about available diagnostics and promoting collaboration between academic institutions and regional healthcare facilities could facilitate knowledge transfer and strengthen overall diagnostic capabilities. In addition, policies that incentivize healthcare professionals to practice in rural or underserved regions could help narrow the gap in access to essential diagnostic services.

In conclusion, while diagnostic tools like microscopy are universally employed in Peru, advanced methods, such as antibody and antigen detection or species identification tests, are limited, particularly outside the capital. Imaging techniques are widely used, but surgical access varied. Antifungal access is uneven, with significant limitations in the availability of lipid formulations of amphotericin B, echinocandins, and flucytosine. Only one center performs TDM, underscoring a critical gap. Enhanced surveillance, equitable access to advanced diagnostics and medications, and implementation of therapeutic drug monitoring are essential to improve IFI management in Peru.

## Data Availability

Data will be shared upon reasonable request after contacting the corresponding author.

## SUPPLEMENTAL MATERIAL

Supplemental tables and figure spectrum.02020-24-s0001.docx Tables S1 and S2, and Fig. S1.

ASM does not own the copyrights to Supplemental Material that may be linked to, or accessed through, an article. The authors have granted ASM a non-exclusive, world-wide license to publish the Supplemental Material files. Please contact the corresponding author directly for reuse.
