# Supplementary material for: Fungi under fire: diagnostic capacities and antifungal availability in Peruvian healthcare facilities
Source: Microbiol Spectr. 2025 Jun 20;13(8):e02020-24. doi: 10.1128/spectrum.02020-24 (PMC12323309; doi:10.1128/spectrum.02020-24)
Supplement: Supplemental tables and figure — Tables S1 and S2, and Fig. S1. [file spectrum.02020-24-s0001.docx]

**FUNGI UNDER FIRE: DIAGNOSTIC CAPACITIES AND ANTIFUNGAL AVAILABILITY IN PERUVIAN HEALTHCARE FACILITIES**

**APPENDIX**

**Supplementary table 1.** Survey answered by participating institutions.

1. ***Institution Profile***
   1. Your position
      1. Attending Physician
      2. Attending Physician - Infectious Diseases Specialist
      3. Clinical microbiologist
      4. Director
      5. Infection Control Practitioner
      6. Laboratory Professional
      7. Professor
      8. Other
   2. Contact information
      1. Your name
      2. Your e-mail address
   3. Institution
      1. Institute
      2. Department
   4. Location of your institution
      1. City
      2. Region/State
      3. Country
   5. Institution profile
      1. Day-Hospital
      2. Dialysis Clinic
      3. Federal Institute / Research Hospital
      4. Oncology Clinic
      5. Private Hospital
      6. Private Laboratory
      7. Public Hospital
      8. University Hospital
      9. Other. Please, specify:
   6. Institution size - number of beds
      1. Overall
      2. Adult intensive care beds
      3. Paediatric/Neonatal intensive care beds
   7. Does your institution take care of patients with any of the following conditions? *Please answer each question with yes, no or unknown.*
      1. COVID-19
      2. Diabetes mellitus
      3. Hematology
      4. HIV/AIDS
      5. Neonatal Intensive Care Unit
      6. Oncology
      7. Parenteral nutrition
      8. Solid organ transplantation
      9. Stem cell transplantation
   8. Does your institution have a microbiology laboratory?
      1. Yes, in place
      2. Yes, outsourcing laboratory services
      3. No
   9. Where is diagnostic mycological procedure performed?
      1. Always in our institution
      2. Part in our institution / part outsourced
      3. Totally outsourced
      4. We do not have access to mycological diagnostic tools
2. ***Perceptions on invasive fungal disease in your institution***
   1. Please rate the incidence of invasive fungal infections in your institution from very low (1) to very high (5)
   2. Please rate the incidence of mucormycosis in your institution from very low (1) to very high (5)
   3. Pathogens of highest importance
      1. *Aspergillus* spp.
      2. *Candida* spp.
      3. *Cryptococcus* spp.
      4. *Fusarium* spp.
      5. *Histoplasma* spp.
      6. Mucorales
   4. What is the approximate number of samples (per month) processed in your mycology laboratory?
      1. TOTAL number of samples
      2. BLOOD samples
      3. BAL (bronchoalveolar lavage) samples
      4. TISSUE (from biopsies) samples
      5. URINE samples
   5. Please indicate all available drugs for antifungal treatment in your institution. *Please answer each question with yes, no or unknown.*
      1. Amphotericin B deoxycholate
      2. Amphotericin B lipid complex
      3. Amphotericin B liposomal
      4. Amphotericin B - other formulations
      5. Anidulafungin
      6. Caspofungin
      7. Fluconazole
      8. Flucytosine (5-FC)
      9. Isavuconazole
      10. Itraconazole
      11. Micafungin
      12. Posaconazole
      13. Terbinafine
      14. Voriconazole
3. ***Microscopy***
   1. Which methodologies are used in fungal microscopy? *Please answer each question with yes, no or unknown.*
      1. Calcofluor white
      2. Giemsa stain
      3. China/India ink
      4. Potassium hydroxide
      5. Silver stain
      6. Others
   2. How frequently is microscopy performed when a fungal disease is suspected from never (1) to always (5)? (e.g., in sterile clinical samples or BAL)
   3. Do you have access to fluorescence dyes?
   4. When cryptococcosis is suspected is direct examination in body fluids available?
      1. Yes, India ink
      2. Yes, other dyes
      3. No
   5. When pneumocystosis is suspected is silver stain performed?
      1. Yes
      2. No
   6. When mucormycosis is suspected, is direct microscopy with optical brighteners performed?
      1. Yes
      2. No
4. ***Culture and Fungal Identification***
   1. Are automated blood cultures available in case of fungemia suspicion?
      1. Yes
      2. No
   2. Please mark all methods used for fungal cultures. *Please answer each question with yes, no or unknown.*
      1. Agar Niger
      2. Chromogen
      3. Lactrimel Agar
      4. Potato Dextrose Agar
      5. Sabouraud
      6. Sabouraud + Chloramphenicol
      7. Sabouraud + Gentamicin
      8. Selective agar (Chloramphenicol + Cycloheximide)
      9. Others
   3. Please select all available test for species identification. *Please answer each question with yes, no or unknown.*
      1. Automated identification (i.e., VITEK, other commercial tests)
      2. Biochemical tests (classic mycology)
      3. DNA sequencing
      4. MALDI – TOF – MS
      5. Mounting medium
   4. Do you have access to antifungal susceptibility tests?
      1. For yeasts
      2. For moulds
      3. For both
      4. None
   5. Which of the following technologies for susceptibility testing are available? *Please answer each question with yes, no or unknown.*
      1. Broth microdilution, using CLSI standards
      2. Broth microdilution, using EUCAST standards
      3. Etest ^®^
      4. VITEK ^®^
   6. Please choose the answer that best matches the maximum identification capability (of yeasts) in your laboratory
      1. Genus
      2. Genus / species
      3. Genus / species / complex
      4. Genus / species / complex / cryptic species
   7. Please choose the answer that best matches the maximum identification capability (of moulds) in your laboratory
      1. Genus
      2. Genus / species
5. ***Serology***
   1. Which of the following serology tests (antibody detection) are available? *Please answer each question with yes, only at an outsourced laboratory, no or unknown.*
      1. *Aspergillus* spp.
      2. *Candida* spp.
      3. *Histoplasma* spp.
6. ***Antigen Detection***
   1. Which of the following antigen detection tests are available? *Please answer each question with yes, only at an outsourced laboratory, no or unknown.*
      1. *Aspergillus* (lateral flow device)
      2. *Aspergillus* galactomannan (immunoenzymatic sandwich microplate assay)
      3. *Aspergillus* galactomannan (lateral flow assay)
      4. *Candida* antigen
      5. *Cryptococcus* (lateral flow assay)
      6. *Cryptococcus* (latex agglutination test)
      7. *Histoplasma*
      8. Beta-d-glucan
7. ***Molecular Tests***
   1. Which of the following molecular tests are available? *Please answer each question with yes, only at an outsourced laboratory, no or unknown.*
      1. *Aspergillus* PCR
      2. *Candida* PCR
      3. *Pneumocystis* PCR
      4. Mucorales PCR
      5. PCR for other fungi
      6. Other molecular tests

1. ***Therapeutic Drug Monitoring (TDM)***
   1. Does your institution have access to therapeutic drug monitoring of antifungal agents? *Please answer each question with yes, only at an outsourced laboratory, no or unknown.*
      1. 5-flucytosine
      2. Itraconazole
      3. Posaconazole
      4. Voriconazole

**Supplementary table 2.** Access to diagnostic and treatment tools of participating institutions in Peru per (a) geographical distribution of the centres (Lima and Callao [capital area, urban] and rest of the country [rural]), (b) climatic distribution (coast line, highlands, and rainforest), (c) healthcare provider (ESSALUD [Seguro Social de Salud - Social Health Insurance] and MINSA [Ministerio de Salud – Ministry of Health] ), and (d) admission of high-risk immunocompromised patients

|  | **Callao and Lima** | | **Provinces** | | **p value** | **Coast** | | **Highlands** | | **Rainforest** | | **p value** | **ESSALUD** | | **MINSA** | | **Army** | | **p value** | **HSCT/SOT** | | **No HSCT/SOT** | | **p value** |
| --- | --- | --- | --- | --- | --- | --- | --- | --- | --- | --- | --- | --- | --- | --- | --- | --- | --- | --- | --- | --- | --- | --- | --- | --- |
|  | *n* | *%* | *n* | *%* |  | *n* | *%* | *N* | *%* | *n* | *%* |  | *n* | *%* | *n* | *%* | *n* | *%* |  | *n* | *%* | *n* | *%* |  |
| **Estimated IFI incidence** | | | | | | | | | | | | | | | | | | | | | | | | |
| Very low | 12/24 | 50.0 | 14/30 | 46.7 | 0.552 | 15/36 | 41.7 | 9/16 | 56.3 | 2/2 | 100.0 | 0.720 | 9/19 | 47.4 | 15/33 | 45.5 | 2/2 | 100.0 | 0.813 | 0/8 | 0.0 | 26/45 | 57.8 | 0.001 |
| Low | 6/24 | 25.0 | 11/30 | 36.7 |  | 13/36 | 36.1 | 4/16 | 25.0 | 0/2 | 0.0 |  | 7/19 | 36.8 | 10/33 | 30.3 | 0/2 | 0.0 |  | 4/8 | 50.0 | 13/45 | 28.9 |  |
| Moderate | 6/24 | 25.0 | 5/30 | 16.7 |  | 8/36 | 22.2 | 3/16 | 18.8 | 0/2 | 0.0 |  | 3/19 | 15.8 | 8/33 | 24.2 | 0/2 | 0.0 |  | 4/8 | 50.0 | 6/45 | 13.3 |  |
| High | 0/24 | 0.0 | 0/30 | 0.0 |  | 0/36 | 0.0 | 0/16 | 0.0 | 0/2 | 0.0 |  | 0/19 | 0.0 | 0/33 | 0.0 | 0/2 | 0.0 |  | 0/8 | 0.0 | 0/45 | 0.0 |  |
| Very high | 0/24 | 0.0 | 0/30 | 0.0 |  | 0/36 | 0.0 | 0/16 | 0.0 | 0/2 | 0.0 |  | 0/19 | 0.0 | 0/33 | 0.0 | 0/2 | 0.0 |  | 0/8 | 0.0 | 0/45 | 0.0 |  |
| **Most relevant pathogen(s) within institutions** | | | | | | | | | | | | | | | | | | | | | | | | |
| *Aspergillus* spp. | 15/24 | 62.5 | 16/30 | 53.3 | 0.585 | 23/36 | 63.9 | 8/16 | 50.0 | 0/2 | 0.0 | 0.143 | 9/19 | 47.4 | 20/33 | 60.6 | 2/2 | 100.0 | 0.397 | 6/8 | 75.0 | 24/45 | 53.3 | 0.441 |
| *Candida* spp. | 23/24 | 95.8 | 27/30 | 90.0 | 0.620 | 33/36 | 91.7 | 15/16 | 93.8 | 2/2 | 100.0 | 1.000 | 17/19 | 89.5 | 31/33 | 93.9 | 2/2 | 100.0 | 0.617 | 8/8 | 100.0 | 41/45 | 91.1 | 1.000 |
| *Cryptococcus* spp. | 15/24 | 62.5 | 16/30 | 53.3 | 0.585 | 23/36 | 63.9 | 6/16 | 37.5 | 1/2 | 50.0 | 0.116 | 6/19 | 31.6 | 23/33 | 69.7 | 1/2 | 50.0 | 0.008 | 3/8 | 37.5 | 26/45 | 57.8 | 0.272 |
| *Histoplasma* spp. | 8/24 | 33.3 | 6/30 | 20.0 | 0.353 | 10/36 | 27.8 | 4/16 | 25.0 | 0/2 | 0.0 | 1.000 | 4/19 | 21.1 | 10/33 | 30.3 | 0/2 | 0.0 | 0.534 | 2/8 | 25.0 | 12/45 | 26.7 | 1.000 |
| Mucorales | 8/24 | 33.3 | 3/30 | 10.0 | 0.046 | 11/36 | 30.6 | 0/16 | 0.0 | 0/2 | 0.0 | 0.024 | 3/19 | 15.8 | 8/33 | 24.2 | 0/2 | 0.0 | 0.726 | 2/8 | 25.0 | 8/45 | 17.8 | 0.636 |
| **Acces to antifungal treatment** | | | | | | | | | | | | | | | | | | | | | | | | |
| AMB | 20/23 | 87.0 | 27/30 | 90.0 | 1.000 | 30/35 | 85.7 | 15/16 | 93.8 | 2/2 | 100.0 | 0.726 | 17/19 | 89.5 | 28/32 | 87.5 | 2/2 | 100.0 | 1.000 | 8/8 | 100.0 | 38/44 | 86.4 | 0.573 |
| *AMB deoxycholate* | 19/23 | 82.6 | 26/30 | 86.7 | 0.715 | 29/35 | 82.9 | 14/16 | 87.5 | 2/2 | 100.0 | 0.010 | 17/19 | 89.5 | 26/32 | 81.3 | 2/2 | 100.0 | 0.694 | 7/8 | 87.5 | 37/44 | 84.1 | 1.000 |
| *AMB lipid complex* | 1/23 | 4.3 | 1/30 | 3.3 | 1.000 | 1/35 | 2.9 | 1/16 | 6.3 | 0/2 | 0.0 | 0.568 | 0/19 | 0.0 | 2/32 | 6.3 | 0/2 | 0.0 | 0.523 | 1/8 | 12.5 | 1/44 | 2.3 | 0.287 |
| *AMB liposomal* | 5/23 | 21.7 | 2/30 | 6.7 | 0.218 | 6/35 | 17.1 | 1/16 | 6.3 | 0/2 | 0.0 | 0.557 | 2/19 | 10.5 | 3/32 | 9.4 | 2/2 | 100.0 | 1.000 | 4/8 | 50.0 | 3/44 | 6.8 | 0.007 |
| Echinocandins | 11/24 | 45.8 | 9/30 | 30.0 | 0.268 | 16/36 | 44.4 | 3/16 | 18.8 | 1/2 | 50.0 | 0.175 | 11/19 | 57.9 | 7/33 | 21.2 | 2/2 | 100.0 | 0.014 | 8/8 | 100.0 | 11/45 | 24.4 | <0.001 |
| *Anidulafungin* | 4/24 | 16.7 | 0/30 | 0.0 | 0.034 | 4/36 | 11.1 | 0/16 | 0.0 | 0/2 | 0.0 | 0.400 | 1/19 | 5.3 | 3/33 | 9.1 | 0/2 | 0.0 | 1.000 | 4/8 | 50.0 | 0/45 | 0.0 | <0.001 |
| *Caspofungin* | 9/23 | 39.1 | 9/30 | 30.0 | 0.565 | 14/35 | 40.0 | 3/16 | 18.8 | 1/2 | 50.0 | 0.293 | 11/19 | 57.9 | 5/32 | 15.6 | 2/2 | 100.0 | 0.004 | 6/8 | 75.0 | 11/44 | 25.0 | 0.011 |
| *Micafungin* | 0/24 | 0.0 | 0/30 | 0.0 | 1.000 | 0/36 | 0.0 | 0/16 | 0.0 | 0/2 | 0.0 | 1.000 | 0/19 | 0.0 | 0/33 | 0.0 | 0/2 | 0.0 | 1.000 | 0/8 | 0.0 | 0/45 | 0.0 | 1.000 |
| Triazoles | 22/24 | 91.7 | 30/30 | 100.0 | 0.193 | 34/36 | 94.4 | 16/16 | 100.0 | 2/2 | 100.0 | 1.000 | 19/19 | 100.0 | 31/33 | 93.9 | 2/2 | 100.0 | 0.527 | 8/8 | 100.0 | 43/45 | 95.6 | 1.000 |
| *Fluconazole* | 22/24 | 91.7 | 29/30 | 96.7 | 0.579 | 33/36 | 91.7 | 16/16 | 100.0 | 2/2 | 100.0 | 0.594 | 19/19 | 100.0 | 30/33 | 90.9 | 2/2 | 100.0 | 0.291 | 8/8 | 100.0 | 42/45 | 93.3 | 1.000 |
| *Isavuconazole* | 7/24 | 29.2 | 0/30 | 0.0 | 0.002 | 7/36 | 19.4 | 0/16 | 0.0 | 0/2 | 0.0 | 0.161 | 0/19 | 0.0 | 5/33 | 15.2 | 2/2 | 100.0 | 0.145 | 3/8 | 37.5 | 3/45 | 6.7 | 0.038 |
| *Itraconazole* | 15/23 | 65.2 | 25/30 | 83.3 | 0.198 | 26/35 | 74.3 | 12/16 | 75.0 | 2/2 | 100.0 | 1.000 | 16/19 | 84.2 | 23/32 | 71.9 | 1/2 | 50.0 | 0.497 | 6/8 | 75.0 | 33/44 | 75.0 | 1.000 |
| *Posaconazole* | 6/23 | 26.1 | 4/30 | 13.3 | 0.300 | 9/35 | 25.7 | 1/16 | 6.3 | 0/2 | 0.0 | 0.201 | 5/19 | 26.3 | 3/32 | 9.4 | 2/2 | 100.0 | 0.131 | 5/7 | 71.4 | 4/45 | 8.9 | <0.001 |
| *Voriconazole* | 12/23 | 52.2 | 7/30 | 23.3 | 0.044 | 16/35 | 45.7 | 3/16 | 18.8 | 0/2 | 0.0 | 0.117 | 9/19 | 47.4 | 8/32 | 25.0 | 2/2 | 100.0 | 0.131 | 7/7 | 100.0 | 11/45 | 24.4 | <0.001 |
| Flucytosine | 0/24 | 0.0 | 0/30 | 0.0 | 1.000 | 0/36 | 0.0 | 0/16 | 0.0 | 0/2 | 0.0 | 1.000 | 0/19 | 0.0 | 0/33 | 0.0 | 0/2 | 0.0 | 1.000 | 0/8 | 0.0 | 0/45 | 0.0 | 1.000 |
| Terbinafine | 9/21 | 42.9 | 9/29 | 31.0 | 0.551 | 14/32 | 43.8 | 2/16 | 12.5 | 2/2 | 100.0 | 0.012 | 6/18 | 33.3 | 11/30 | 36.7 | 1/2 | 50.0 | 1.000 | 2/6 | 33.3 | 15/43 | 34.9 | 1.000 |
| **Microscopy** | 24/24 | 100.0 | 30/30 | 100.0 | 1.000 | 36/36 | 100.0 | 16/16 | 100.0 | 2/2 | 100.0 | 1.000 | 19/19 | 100.0 | 33/33 | 100.0 | 2/2 | 100.0 | 1.000 | 8/8 | 100.0 | 45/45 | 100.0 | 1.000 |
| Microscopy use if IFI suspected |  |  |  |  |  |  |  |  |  |  |  |  |  |  |  |  |  |  |  |  |  |  |  |  |
| *Never* | 5/24 | 20.8 | 9/30 | 30.0 | 0.792 | 9/36 | 25.0 | 5/16 | 31.3 | 0/2 | 0.0 | 0.531 | 7/19 | 36.8 | 7/33 | 21.2 | 0/2 | 0.0 | 0.570 | 1/8 | 12.5 | 13/45 | 28.9 | 0.281 |
| *Almost never* | 7/24 | 29.2 | 10/30 | 33.3 |  | 10/36 | 27.8 | 6/16 | 37.5 | 1/2 | 50.0 |  | 4/19 | 21.1 | 12/33 | 36.4 | 1/2 | 50.0 |  | 2/8 | 25.0 | 15/45 | 33.3 |  |
| *Sometimes* | 5/24 | 20.8 | 6/30 | 20.0 |  | 7/36 | 19.4 | 4/16 | 25.0 | 0/2 | 0.0 |  | 4/19 | 21.1 | 7/33 | 21.2 | 0/2 | 0.0 |  | 3/8 | 37.5 | 7/45 | 15.6 |  |
| *Almost always* | 3/24 | 12.5 | 3/30 | 10.0 |  | 5/36 | 13.9 | 1/16 | 6.3 | 0/2 | 0.0 |  | 1/19 | 5.3 | 4/33 | 12.1 | 1/2 | 50.0 |  | 0/8 | 0.0 | 6/45 | 13.3 |  |
| *Always* | 4/24 | 16.7 | 2/30 | 6.7 |  | 5/36 | 13.9 | 0/16 | 0.0 | 1/2 | 50.0 |  | 3/19 | 15.8 | 3/33 | 9.1 | 0/2 | 0.0 |  | 2/8 | 25.0 | 4/45 | 8.9 |  |
| Calcofluor white | 1/9 | 11.1 | 0/13 | 0.0 | 0.409 | 1/15 | 6.7 | 0/7 | 0.0 | 0/0 | 0.0 | 1.000 | 0/7 | 0.0 | 1/14 | 7.1 | 0/1 | 0.0 | 1.000 | 0/4 | 0.0 | 1/18 | 5.6 | 1.000 |
| Giemsa stain | 16/21 | 76.2 | 23/28 | 82.1 | 0.726 | 26/33 | 78.8 | 11/14 | 78.6 | 2/2 | 100.0 | 1.000 | 15/17 | 88.2 | 22/30 | 73.3 | 2/2 | 100.0 | 0.289 | 4/7 | 57.1 | 34/41 | 82.9 | 0.147 |
| China/India ink | 23/24 | 95.8 | 24/28 | 85.7 | 0.358 | 34/36 | 94.4 | 11/14 | 78.6 | 2/2 | 100.0 | 0.288 | 14/18 | 77.8 | 31/32 | 96.9 | 2/2 | 100.0 | 0.050 | 8/8 | 100.0 | 38/43 | 88.4 | 0.580 |
| KOH | 19/20 | 95.0 | 25/30 | 83.3 | 0.381 | 27/32 | 84.4 | 15/16 | 93.8 | 2/2 | 100.0 | 0.728 | 17/18 | 94.4 | 26/31 | 83.9 | 1/1 | 100.0 | 0.393 | 5/6 | 83.3 | 38/43 | 88.4 | 0.564 |
| Silver stain | 7/17 | 41.2 | 4/19 | 21.1 | 0.281 | 8/26 | 30.8 | 2/9 | 22.2 | 1/1 | 100.0 | 0.456 | 4/13 | 30.8 | 6/22 | 27.3 | 1/1 | 100.0 | 1.000 | 2/5 | 40.0 | 8/30 | 26.7 | 0.610 |
| Fluorescence dyes | 2/24 | 8.3 | 1/30 | 3.3 | 0.579 | 3/36 | 8.3 | 0/16 | 0.0 | 0/2 | 0.0 | 0.594 | 0/19 | 0.0 | 3/33 | 9.1 | 0/2 | 0.0 | 0.291 | 0/8 | 0.0 | 3/45 | 6.7 | 1.000 |
| **Culture** | 21/21 | 100.0 | 23/28 | 82.1 | 0.062 | 29/32 | 90.6 | 13/15 | 86.7 | 2/2 | 100.0 | 0.717 | 15/17 | 88.2 | 28/31 | 90.3 | 1/1 | 100.0 | 1.000 | 6/7 | 85.7 | 37/41 | 90.2 | 0.562 |
| Blood culture if fungemia suspected | 17/21 | 81.0 | 12/28 | 42.9 | 0.009 | 22/32 | 68.8 | 6/15 | 40.0 | 1/2 | 50.0 | 0.152 | 7/17 | 41.2 | 21/31 | 67.7 | 1/1 | 100.0 | 0.125 | 6/7 | 85.7 | 22/41 | 53.7 | 0.214 |
| Available media for fungal culture |  |  |  |  |  |  |  |  |  |  |  |  |  |  |  |  |  |  |  |  |  |  |  |  |
| *Agar Niger* | 3/8 | 37.5 | 1/18 | 5.6 | 0.072 | 4/16 | 25.0 | 0/9 | 0.0 | 0/1 | 0.0 | 0.374 | 1/10 | 10.0 | 3/16 | 18.8 | 0/0 | 0.0 | 1.000 | 1/3 | 33.3 | 3/23 | 13.0 | 0.408 |
| *Chromogen agar* | 7/15 | 46.7 | 1/20 | 5.0 | 0.011 | 7/23 | 30.4 | 1/11 | 9.1 | 0/1 | 0.0 | 0.404 | 1/13 | 7.7 | 6/21 | 28.6 | 1/1 | 100.0 | 0.210 | 3/5 | 60.0 | 4/29 | 13.8 | 0.048 |
| *Lactrimel agar* | 0/7 | 0.0 | 1/20 | 5.0 | 1.000 | 1/15 | 6.7 | 0/11 | 0.0 | 0/1 | 0.0 | 1.000 | 1/12 | 8.3 | 0/15 | 0.0 | 0/0 | 0.0 | 0.444 | 0/3 | 0.0 | 1/24 | 4.2 | 1.000 |
| *Potato agar* | 3/9 | 33.3 | 2/21 | 9.5 | 0.143 | 4/18 | 22.2 | 1/10 | 10.0 | 0/2 | 0.0 | 0.742 | 2/12 | 16.7 | 3/18 | 16.7 | 0/0 | 0.0 | 1.000 | 1/3 | 33.3 | 4/27 | 14.8 | 0.433 |
| *Sabouraud* | 17/18 | 94.4 | 21/26 | 80.8 | 0.375 | 24/28 | 85.7 | 13/14 | 92.9 | 1/2 | 50.0 | 0.288 | 14/16 | 87.5 | 23/27 | 85.2 | 1/1 | 100.0 | 1.000 | 5/6 | 83.3 | 32/37 | 86.5 | 1.000 |
| *Sabouraud + chloramphenicol* | 5/9 | 55.6 | 7/20 | 35.0 | 0.422 | 8/19 | 42.1 | 4/9 | 44.4 | 0/1 | 0.0 | 1.000 | 2/10 | 20.0 | 9/18 | 50.0 | 1/1 | 100.0 | 0.226 | 3/5 | 60.0 | 9/24 | 37.5 | 0.622 |
| *Sabouraud + gentamicin* | 3/7 | 42.9 | 5/19 | 26.3 | 0.635 | 6/16 | 37.5 | 2/9 | 22.2 | 0/1 | 0.0 | 0.765 | 1/9 | 11.1 | 7/17 | 41.2 | 0/0 | 0.0 | 0.190 | 2/5 | 40.0 | 6/21 | 28.6 | 0.628 |
| *Selective agar (chloramphenicol + gentamicin)* | 1/5 | 20.0 | 3/19 | 15.8 | 1.000 | 3/14 | 21.4 | 1/9 | 11.1 | 0/1 | 0.0 | 1.000 | 1/9 | 11.1 | 3/15 | 20.0 | 0/0 | 0.0 | 1.000 | 0/3 | 0.0 | 4/21 | 19.0 | 1.000 |
| Available tests for species identification | 19/21 | 90.5 | 18/28 | 64.3 | 0.047 | 27/32 | 84.4 | 8/15 | 53.3 | 2/2 | 100.0 | 0.067 | 12/17 | 70.6 | 24/31 | 77.4 | 1/1 | 100.0 | 0.731 | 5/7 | 71.4 | 31/41 | 75.6 | 1.000 |
| *Semi-automated identification kit* | 14/19 | 73.7 | 12/26 | 46.2 | 0.077 | 21/28 | 75.0 | 4/15 | 26.7 | 1/2 | 50.0 | 0.003 | 9/17 | 52.9 | 17/28 | 60.7 | 0/0 | 0.0 | 0.757 | 4/6 | 66.7 | 21/38 | 55.3 | 0.684 |
| *Automated identification system* | 14/15 | 93.3 | 14/25 | 56.0 | 0.030 | 19/25 | 76.0 | 7/13 | 53.8 | 2/2 | 100.0 | 0.274 | 8/13 | 61.5 | 19/26 | 73.1 | 1/1 | 100.0 | 0.486 | 5/6 | 83.3 | 22/33 | 66.7 | 0.645 |
| *DNA sequencing* | 0/19 | 0.0 | 2/28 | 7.1 | 0.508 | 1/30 | 3.3 | 1/15 | 6.7 | 0/2 | 0.0 | 1.000 | 1/17 | 5.9 | 1/29 | 3.4 | 0/1 | 0.0 | 1.000 | 0/6 | 0.0 | 2/40 | 5.0 | 1.000 |
| *MALDI-TOF-MS* | 1/19 | 5.3 | 0/27 | 0.0 | 0.413 | 1/29 | 3.4 | 0/15 | 0.0 | 0/2 | 0.0 | 1.000 | 0/17 | 0.0 | 1/28 | 3.6 | 0/1 | 0.0 | 1.000 | 1/5 | 20.0 | 0/40 | 0.0 | 0.111 |
| Antifungal susceptibility | 13/21 | 61.9 | 14/28 | 50.0 | 0.563 | 21/32 | 65.6 | 5/15 | 33.3 | 1/2 | 50.0 | 0.088 | 7/17 | 41.2 | 19/31 | 61.3 | 1/1 | 100.0 | 0.232 | 5/7 | 71.4 | 21/41 | 51.2 | 0.429 |
| *CLSI* | 7/15 | 46.7 | 4/25 | 16.0 | 0.065 | 10/25 | 40.0 | 0/14 | 0.0 | 1/1 | 100.0 | 0.002 | 3/15 | 20.0 | 7/24 | 29.2 | 1/1 | 100.0 | 0.711 | 3/4 | 75.0 | 8/36 | 22.2 | 0.056 |
| *EUCAST* | 1/13 | 7.7 | 0/25 | 0.0 | 0.342 | 1/23 | 4.3 | 0/14 | 0.0 | 0/1 | 0.0 | 1.000 | 0/15 | 0.0 | 1/23 | 4.3 | 0/0 | 0.0 | 1.000 | 0/4 | 0.0 | 1/34 | 2.9 | 1.000 |
| *Gradient strip test* | 3/12 | 25.0 | 2/24 | 8.3 | 0.307 | 5/21 | 23.8 | 0/14 | 0.0 | 0/1 | 0.0 | 0.198 | 2/15 | 13.3 | 2/20 | 10.0 | 1/1 | 100.0 | 1.000 | 2/4 | 50.0 | 3/32 | 9.4 | 0.084 |
| *Semiautomated antifungal susceptibility testing system* | 3/14 | 21.4 | 10/25 | 40.0 | 0.304 | 10/25 | 40.0 | 2/13 | 15.4 | 1/1 | 100.0 | 0.071 | 5/15 | 33.3 | 8/23 | 34.8 | 0/1 | 0.0 | 1.000 | 0/4 | 0.0 | 13/35 | 37.1 | 0.281 |
| **Antibody detection** | 11/20 | 55.0 | 3/27 | 11.1 | 0.003 | 12/31 | 38.7 | 2/15 | 13.3 | 0/1 | 0.0 | 0.159 | 3/16 | 18.8 | 10/30 | 33.3 | 1/1 | 100.0 | 0.493 | 4/7 | 57.1 | 9/39 | 23.1 | 0.087 |
| *Aspergillus* spp. | 8/20 | 40.0 | 1/26 | 3.8 | 0.006 | 9/31 | 29.0 | 0/14 | 0.0 | 0/1 | 0.0 | 0.049 | 1/15 | 6.7 | 7/30 | 23.3 | 1/1 | 100.0 | 0.236 | 4/7 | 57.1 | 5/38 | 13.2 | 0.022 |
| *Onsite* | 1/8 | 12.5 | 0/1 | 0.0 |  | 1/9 | 11.1 | 0/0 | 0.0 | 0/0 | 0.0 |  | 1/1 | 100.0 | 0/7 | 0.0 | 0/1 | 0.0 |  | 1/4 | 25.0 | 0/5 | 0.0 |  |
| *Outsourced* | 7/8 | 87.5 | 1/1 | 100.0 |  | 8/9 | 88.9 | 0/0 | 0.0 | 0/0 | 0.0 |  | 0/1 | 0.0 | 7/7 | 100.0 | 1/1 | 100.0 |  | 3/4 | 75.0 | 5/5 | 100.0 |  |
| *Candida* spp. | 7/20 | 35.0 | 2/26 | 7.7 | 0.029 | 8/31 | 25.8 | 1/14 | 7.1 | 0/1 | 0.0 | 0.385 | 1/15 | 6.7 | 7/30 | 23.3 | 1/1 | 100.0 | 0.236 | 3/7 | 42.9 | 6/38 | 15.8 | 0.131 |
| *Onsite* | 1/7 | 14.3 | 2/2 | 100.0 |  | 2/8 | 25.0 | 1/1 | 100.0 | 0/0 | 0.0 |  | 1/1 | 100.0 | 2/7 | 28.6 | 0/1 | 0.0 |  | 1/3 | 33.3 | 2/6 | 33.3 |  |
| *Outsourced* | 6/7 | 85.7 | 0/2 | 0.0 |  | 6/8 | 75.0 | 0/1 | 0.0 | 0/0 | 0.0 |  | 0/1 | 0.0 | 5/7 | 71.4 | 1/1 | 100.0 |  | 2/3 | 66.7 | 4/6 | 66.7 |  |
| *Histoplasma* spp. | 11/20 | 55.0 | 2/26 | 7.7 | <0.001 | 12/31 | 38.7 | 1/14 | 7.1 | 0/1 | 0.0 | 0.047 | 3/16 | 18.8 | 9/29 | 31.0 | 1/1 | 100.0 | 0.491 | 4/7 | 57.1 | 8/38 | 21.1 | 0.069 |
| *Outsourced* | 11/11 | 100.0 | 1/2 | 50.0 |  | 11/12 | 91.7 | 1/1 | 100.0 | 0/0 | 0.0 |  | 3/3 | 100.0 | 8/9 | 88.9 | 1/1 | 100.0 |  | 4/4 | 100.0 | 7/8 | 87.5 |  |
| *Paracoccidioides* spp. | 8/20 | 40.0 | 2/26 | 7.7 | 0.012 | 9/31 | 29.0 | 1/14 | 7.1 | 0/1 | 0.0 | 0.325 | 2/16 | 12.5 | 7/29 | 24.1 | 1/1 | 100.0 | 0.456 | 3/7 | 42.9 | 7/38 | 18.4 | 0.172 |
| *Outsourced* | 8/8 | 100.0 | 2/2 | 100.0 |  | 9/9 | 100.0 | 1/1 | 100.0 | 0/0 | 0.0 |  | 2/2 | 100.0 | 7/7 | 100.0 | 1/1 | 100.0 |  | 3/3 | 100.0 | 7/7 | 100.0 |  |
| **Antigen detection** | 14/21 | 66.7 | 7/25 | 28.0 | 0.017 | 15/30 | 50.0 | 5/15 | 33.3 | 1/1 | 100.0 | 0.346 | 5/15 | 33.3 | 15/30 | 50.0 | 1/1 | 100.0 | 0.352 | 5/7 | 71.4 | 15/38 | 39.5 | 0.214 |
| *Aspergillus* GM, any | 10/21 | 47.6 | 1/25 | 4.0 | 0.001 | 11/30 | 36.7 | 0/15 | 0.0 | 0/1 | 0.0 | 0.011 | 2/15 | 13.3 | 8/30 | 26.7 | 1/1 | 100.0 | 0.456 | 5/7 | 71.4 | 5/38 | 13.2 | 0.003 |
| *Aspergillus* GM (ELISA) | 9/20 | 45.0 | 1/24 | 4.2 | 0.002 | 10/28 | 35.7 | 0/15 | 0.0 | 0/1 | 0.0 | 0.010 | 2/15 | 13.3 | 7/28 | 25.0 | 1/1 | 100.0 | 0.458 | 5/7 | 71.4 | 4/36 | 11.1 | 0.002 |
| *Onsite* | 3/9 | 33.3 | 0/1 | 0.0 |  | 3/10 | 30.0 | 0/0 | 0.0 | 0/0 | 0.0 |  | 1/2 | 50.0 | 2/7 | 28.6 | 0/1 | 0.0 |  | 3/5 | 60.0 | 0/4 | 0.0 |  |
| *Outsourced* | 6/9 | 66.7 | 1/1 | 100.0 |  | 7/10 | 70.0 | 0/0 | 0.0 | 0/0 | 0.0 |  | 1/2 | 50.0 | 5/7 | 71.4 | 1/1 | 100.0 |  | 2/5 | 40.0 | 4/4 | 100.0 |  |
| *Aspergillus* GM (LFA) | 7/20 | 35.0 | 1/24 | 4.2 | 0.015 | 8/28 | 28.6 | 0/15 | 0.0 | 0/1 | 0.0 | 0.043 | 2/15 | 13.3 | 5/28 | 17.9 | 1/1 | 100.0 | 1.000 | 3/7 | 42.9 | 4/36 | 11.1 | 0.072 |
| *Onsite* | 1/7 | 14.3 | 0/1 | 0.0 |  | 1/8 | 12.5 | 0/0 | 0.0 | 0/0 | 0.0 |  | 1/2 | 50.0 | 0/5 | 0.0 | 0/1 | 0.0 |  | 1/3 | 33.3 | 0/4 | 0.0 |  |
| *Outsourced* | 6/7 | 85.7 | 1/1 | 100.0 |  | 7/8 | 87.5 | 0/0 | 0.0 | 0/0 | 0.0 |  | 1/2 | 50.0 | 5/5 | 100.0 | 1/1 | 100.0 |  | 2/3 | 66.7 | 4/4 | 100.0 |  |
| *Aspergillus* GM (LFD) | 6/21 | 28.6 | 0/24 | 0.0 | 0.007 | 6/29 | 20.7 | 0/15 | 0.0 | 0/1 | 0.0 | 0.203 | 0/15 | 0.0 | 5/29 | 17.2 | 1/1 | 100.0 | 0.149 | 2/7 | 28.6 | 4/37 | 10.8 | 0.238 |
| *Outsourced* | 6/6 | 100.0 | 0/0 | 0.0 |  | 6/6 | 100.0 | 0/0 | 0.0 | 0/0 | 0.0 |  | 0/0 | 0.0 | 5/5 | 100.0 | 1/1 | 100.0 |  | 2/2 | 100.0 | 4/4 | 100.0 |  |
| *Candida* antimannan | 6/20 | 30.0 | 1/24 | 4.2 | 0.035 | 7/28 | 25.0 | 0/15 | 0.0 | 0/1 | 0.0 | 0.090 | 1/15 | 6.7 | 5/28 | 17.9 | 1/1 | 100.0 | 0.403 | 2/7 | 28.6 | 4/36 | 11.1 | 0.248 |
| *Outsourced* | 6/6 | 100.0 | 1/1 | 100.0 |  | 7/7 | 100.0 | 0/0 | 0.0 | 0/0 | 0.0 |  | 1/1 | 100.0 | 5/5 | 100.0 | 1/1 | 100.0 |  | 2/2 | 100.0 | 4/4 | 100.0 |  |
| *Cryptococcus* mannan, any | 13/21 | 61.9 | 6/24 | 25.0 | 0.017 | 14/29 | 48.3 | 4/15 | 26.7 | 1/1 | 100.0 | 0.203 | 5/15 | 33.3 | 13/29 | 44.8 | 1/1 | 100.0 | 0.531 | 4/7 | 57.1 | 14/37 | 37.8 | 0.419 |
| *Cryptococcus* (LAT) | 13/21 | 61.9 | 6/24 | 25.0 | 0.017 | 14/29 | 48.3 | 4/15 | 26.7 | 1/1 | 100.0 | 0.203 | 5/15 | 33.3 | 13/29 | 44.8 | 1/1 | 100.0 | 0.531 | 4/7 | 57.1 | 14/37 | 37.8 | 0.419 |
| *Onsite* | 5/13 | 38.5 | 3/6 | 50.0 |  | 5/14 | 35.7 | 3/4 | 75.0 | 0/1 | 0.0 |  | 3/5 | 60.0 | 5/13 | 38.5 | 0/1 | 0.0 |  | 3/4 | 75.0 | 5/14 | 35.7 |  |
| *Outsourced* | 8/13 | 61.5 | 3/6 | 50.0 |  | 9/14 | 64.3 | 1/4 | 25.0 | 1/1 | 100.0 |  | 2/5 | 40.0 | 8/13 | 61.5 | 1/1 | 100.0 |  | 1/4 | 25.0 | 9/14 | 64.3 |  |
| *Cryptococcus* (LFA) | 10/21 | 47.6 | 1/23 | 4.3 | 0.001 | 10/29 | 34.5 | 1/14 | 7.1 | 0/1 | 0.0 | 0.116 | 1/15 | 6.7 | 9/28 | 32.1 | 1/1 | 100.0 | 0.127 | 4/7 | 57.1 | 6/36 | 16.7 | 0.040 |
| *Onsite* | 3/10 | 30.0 | 1/1 | 100.0 |  | 3/10 | 30.0 | 1/1 | 100.0 | 0/0 | 0.0 |  | 1/1 | 100.0 | 3/9 | 33.3 | 0/1 | 0.0 |  | 3/4 | 75.0 | 1/6 | 16.7 |  |
| *Outsourced* | 7/10 | 70.0 | 0/1 | 0.0 |  | 7/10 | 70.0 | 0/1 | 0.0 | 0/0 | 0.0 |  | 0/1 | 0.0 | 6/9 | 66.7 | 1/1 | 100.0 |  | 1/4 | 25.0 | 5/6 | 83.3 |  |
| *Histoplasma* antigen | 9/21 | 42.9 | 4/23 | 17.4 | 0.099 | 10/29 | 34.5 | 2/14 | 14.3 | 1/1 | 100.0 | 0.137 | 2/14 | 14.3 | 10/29 | 34.5 | 1/1 | 100.0 | 0.279 | 3/7 | 42.9 | 9/36 | 25.0 | 0.378 |
| *Outsourced* | 9/9 | 100.0 | 4/4 | 100.0 |  | 10/10 | 100.0 | 2/2 | 100.0 | 1/1 | 100.0 |  | 2/2 | 100.0 | 10/10 | 100.0 | 1/1 | 100.0 |  | 3/3 | 100.0 | 9/9 | 100.0 |  |
| Beta-d-glucan | 6/21 | 28.6 | 2/24 | 8.3 | 0.121 | 7/29 | 24.1 | 1/15 | 6.7 | 0/1 | 0.0 | 0.368 | 1/15 | 6.7 | 6/29 | 20.7 | 1/1 | 100.0 | 0.393 | 2/7 | 28.6 | 6/37 | 16.2 | 0.593 |
| *Outsourced* | 6/6 | 100.0 | 2/2 | 100.0 |  | 7/7 | 100.0 | 1/1 | 100.0 | 0/0 | 0.0 |  | 1/1 | 100.0 | 6/6 | 100.0 | 1/1 | 100.0 |  | 2/2 | 100.0 | 6/6 | 100.0 |  |
| **Molecular tests** | 8/20 | 40.0 | 2/24 | 8.3 | 0.027 | 9/28 | 32.1 | 1/15 | 6.7 | 0/1 | 0.0 | 0.152 | 1/15 | 6.7 | 8/28 | 28.6 | 1/1 | 100.0 | 0.129 | 3/7 | 42.9 | 7/37 | 18.9 | 0.322 |
| *Aspergillus* PCR | 6/20 | 30.0 | 1/24 | 4.2 | 0.035 | 7/28 | 25.0 | 0/15 | 0.0 | 0/1 | 0.0 | 0.090 | 1/15 | 6.7 | 5/28 | 17.9 | 1/1 | 100.0 | 0.403 | 3/7 | 42.9 | 4/37 | 10.8 | 0.068 |
| *Onsite* | 1/6 | 16.7 | 1/1 | 100.0 |  | 2/7 | 28.6 | 0/0 | 0.0 | 0/0 | 0.0 |  | 1/1 | 100.0 | 1/5 | 20.0 | 0/1 | 0.0 |  | 1/3 | 33.3 | 1/4 | 25.0 |  |
| *Outsourced* | 5/6 | 83.3 | 0/1 | 0.0 |  | 5/7 | 71.4 | 0/0 | 0.0 | 0/0 | 0.0 |  | 0/1 | 0.0 | 4/5 | 80.0 | 1/1 | 100.0 |  | 2/3 | 66.7 | 3/4 | 75.0 |  |
| *Candida* PCR | 5/20 | 25.0 | 2/24 | 8.3 | 0.217 | 6/28 | 21.4 | 1/15 | 6.7 | 0/1 | 0.0 | 0.488 | 1/15 | 6.7 | 5/28 | 17.9 | 1/1 | 100.0 | 0.403 | 2/7 | 28.6 | 5/37 | 13.5 | 0.307 |
| *Outsourced* | 5/5 | 100.0 | 1/2 | 50.0 |  | 5/6 | 83.3 | 1/1 | 100.0 | 0/0 | 0.0 |  | 0/1 | 0.0 | 5/5 | 100.0 | 1/1 | 100.0 |  | 2/2 | 100.0 | 4/5 | 80.0 |  |
| *Pneumocystis* PCR | 5/20 | 25.0 | 0/24 | 0.0 | 0.014 | 5/28 | 17.9 | 0/15 | 0.0 | 0/1 | 0.0 | 0.242 | 0/15 | 0.0 | 4/28 | 14.3 | 1/1 | 100.0 | 0.280 | 2/7 | 28.6 | 3/37 | 8.1 | 0.173 |
| *Outsourced* | 5/5 | 100.0 | 0/0 | 0.0 |  | 5/5 | 100.0 | 0/0 | 0.0 | 0/0 | 0.0 |  | 0/0 | 0.0 | 4/4 | 100.0 | 1/1 | 100.0 |  | 2/2 | 100.0 | 3/3 | 100.0 |  |
| Mucorales PCR | 5/20 | 25.0 | 0/24 | 0.0 | 0.014 | 5/28 | 17.9 | 0/15 | 0.0 | 0/1 | 0.0 | 0.242 | 0/15 | 0.0 | 4/28 | 14.3 | 1/1 | 100.0 | 0.280 | 2/7 | 28.6 | 3/37 | 8.1 | 0.173 |
| *Outsourced* | 5/5 | 100.0 | 0/0 | 0.0 |  | 5/5 | 100.0 | 0/0 | 0.0 | 0/0 | 0.0 |  | 0/0 | 0.0 | 4/4 | 100.0 | 1/1 | 100.0 |  | 2/2 | 100.0 | 3/3 | 100.0 |  |
| **Therapeutic drug monitoring** | 1/21 | 4.8 | 0/25 | 0.0 | - | 1/30 | 3.3 | 0/15 | 0.0 | 0/1 | 0.0 | - | 0/15 | 0.0 | 1/30 | 3.3 | 0/1 | 0.0 | - | 1/7 | 14.3 | 0/38 | 0.0 | - |
| Posaconazole | 1/21 | 4.8 | 0/25 | 0.0 | - | 1/30 | 3.3 | 0/15 | 0.0 | 0/1 | 0.0 | - | 0/15 | 0.0 | 1/30 | 3.3 | 0/1 | 0.0 | - | 1/7 | 14.3 | 0/38 | 0.0 | - |
| *Onsite* | 1/1 | 100.0 | 0/0 | 0.0 |  | 1/1 | 100.0 | 0/0 | 0.0 | 0/0 | 0.0 |  | 0/0 | 0.0 | 1/1 | 100.0 | 0/0 | 0.0 |  | 1/1 | 100.0 | 0/0 | 0.0 |  |
| Voriconazole | 1/21 | 4.8 | 0/25 | 0.0 | - | 1/30 | 3.3 | 0/15 | 0.0 | 0/1 | 0.0 | - | 0/15 | 0.0 | 1/30 | 3.3 | 0/1 | 0.0 | - | 1/7 | 14.3 | 0/38 | 0.0 | - |
| *Onsite* | 1/1 | 100.0 | 0/0 | 0.0 |  | 1/1 | 100.0 | 0/0 | 0.0 | 0/0 | 0.0 |  | 0/0 | 0.0 | 1/1 | 100.0 | 0/0 | 0.0 |  | 1/1 | 100.0 | 0/0 | 0.0 |  |
| **Imaging procedures** | | | | | | | | | | | | | | | | | | | | | | | | |
| CT | 17/21 | 81.0 | 22/25 | 88.0 | 0.686 | 25/30 | 83.3 | 13/15 | 86.7 | 1/1 | 100.0 | 1.000 | 12/15 | 80.0 | 26/30 | 86.7 | 1/1 | 100.0 | 0.670 | 6/7 | 85.7 | 32/38 | 84.2 | 1.000 |
| PET CT | 2/21 | 9.5 | 0/25 | 0.0 | 0.203 | 2/30 | 6.7 | 0/15 | 0.0 | 0/1 | 0.0 | 0.565 | 1/15 | 6.7 | 1/30 | 3.3 | 0/1 | 0.0 | 1.000 | 2/7 | 28.6 | 0/38 | 0.0 | 0.021 |
| MRI | 5/21 | 23.8 | 9/25 | 36.0 | 0.522 | 9/30 | 30.0 | 5/15 | 33.3 | 0/1 | 0.0 | 1.000 | 4/15 | 26.7 | 9/30 | 30.0 | 1/1 | 100.0 | 1.000 | 4/7 | 57.1 | 9/38 | 23.7 | 0.168 |
| PET MRI | 2/21 | 9.5 | 0/25 | 0.0 | 0.203 | 2/30 | 6.7 | 0/15 | 0.0 | 0/1 | 0.0 | 0.565 | 1/15 | 6.7 | 1/30 | 3.3 | 0/1 | 0.0 | 1.000 | 2/7 | 28.6 | 0/38 | 0.0 | 0.021 |
| Ultrasound | 20/21 | 95.2 | 23/25 | 92.0 | 1.000 | 28/30 | 93.3 | 14/15 | 93.3 | 1/1 | 100.0 | 1.000 | 14/15 | 93.3 | 28/30 | 93.3 | 1/1 | 100.0 | 1.000 | 7/7 | 100.0 | 35/38 | 92.1 | 1.000 |
| X ray | 18/21 | 85.7 | 19/25 | 76.0 | 0.478 | 24/30 | 80.0 | 12/15 | 80.0 | 1/1 | 100.0 | 1.000 | 11/15 | 73.3 | 25/30 | 83.3 | 1/1 | 100.0 | 0.454 | 7/7 | 100.0 | 29/38 | 76.3 | 0.315 |
| **Surgery** | 13/18 | 72.2 | 5/13 | 38.5 | 0.010 | 15/24 | 62.5 | 3/6 | 50.0 | 0/1 | 0.0 | 0.024 | 2/6 | 33.3 | 15/24 | 62.5 | 1/1 | 100.0 | 0.014 | 5/6 | 83.3 | 12/24 | 50.0 | 0.193 |

AMB, amphotericin B; CLSI, Clinical and Laboratory Standards Institute; CT, computed tomography; DNA, deoxyribonucleic acid; ELISA, enzyme-linked immunosorbent assay; ESSALUD, Seguro Social de Salud - Social Health Insurance; EUCAST, European Committee on Antimicrobial Susceptibility Testing; GM, galactomannan; HSCT, haematopoietic stem-cell transplantation; IFI, invasive fungal infection; KOH, potassium hydroxide; LAT, latex agglutination test; LFA, lateral flow assay; LFD, lateral flow device; MALDI-TOF-MS, matrix-assisted laser desorption/ionization-time of flight mass spectrometry; MINSA, Ministerio de Salud - Ministry of Health; MRI, magnetic resonance imaging; PCR, polymerase chain reaction; PET, positron emission tomography; SOT, solid organ trasnplantation; spp., species

**Supplementary figure 1.** Map of the Peruvian departments (Peruvian term for regions) by climate type


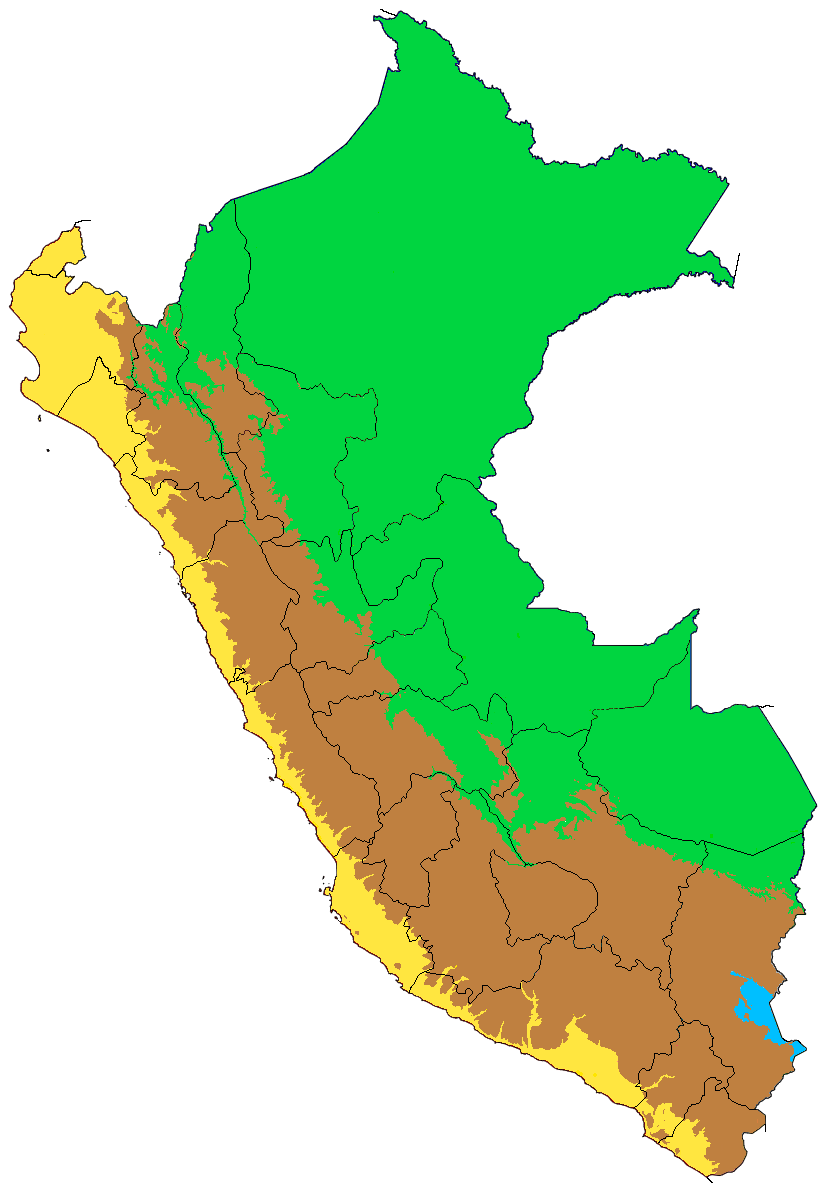


Original source: https://en.wikipedia.org/wiki/Climate_of_Peru

Coastal desert (yellow) – Andes mountains (brown) – Amazon rainforest (green)
